# Improving Nutrition Habits and Reducing Sedentary Time Among Preschool-Aged Children in Cuenca, Ecuador: A Trial of a School-Based Intervention

**DOI:** 10.5888/pcd15.180053

**Published:** 2018-07-26

**Authors:** Matthew L. Romo, Victoria Abril-Ulloa

**Affiliations:** 1 Carrera de Nutrición y Dietética, Facultad de Ciencias Médicas, Universidad de Cuenca, Cuenca, Ecuador; 2 Dirección de Investigación, Universidad de Cuenca, Cuenca, Ecuador

## Abstract

**Introduction:**

In Ecuador, the prevalence of overweight and obesity among school-age children is more than triple that of preschool-age children; however, preschoolers have not been the target of interventions.

**Methods:**

We developed an educational and behavioral intervention that included games, singing, and storytelling. Children were recruited from municipal preschools in Cuenca and were enrolled in the pilot intervention (PI) (N=155) for the 2015–2016 school year, which consisted of a 3-month in-school program. For the 2016–2017 school year, a separate group of children was enrolled in the enhanced intervention (EI) (N=152), which consisted of a 7-month program at both school and home.

**Results:**

Parents in both groups reported a post-intervention reduction in their child’s daily at-home consumption of sugar-sweetened beverages (PI: −23.2%, *P* < .001; EI: −16.8%, *P* < .001). Additional beneficial effects of the EI not observed with the PI were an increase in drinking water daily at home (+8.3%, *P* = .04) and eating fruits and vegetables daily for snacks at home (+21.8%, *P* < .001), a reduction in excessive weekend screen time (−7.6%, *P* = .03), and a reduction of 0.11 in mean BMI-for-age *z *score (*P* = .003). When comparing the PI and EI, the EI was associated with a greater difference in mean BMI-for-age *z *score **(**−0.25; *P* < .001) and fruit and vegetable consumption (+15.9%; *P* = .01).

**Conclusion:**

Our preschool-based intervention appeared to be successful in promoting healthy lifestyle habits, especially when combined with a household component. Further research is needed to determine if the intervention had long-term effects, as well as to adapt it for different settings.

## Introduction

Overweight and obesity are major public health problems facing Ecuador (1). The high prevalence of overweight and obesity among children is particularly concerning, as they will likely experience worse health and more disability throughout their lifetime than their normal-weight peers (2). At a national level in Ecuador, the prevalence of overweight and obesity is 8.6% among preschool-aged children (<5 years) and is more than triple (29.9%) among school-aged children (5–11 years) (3,4). Because young children who are overweight are more likely to become obese in later childhood (5) and because the preschool years are a critical period of formation of healthy lifestyle habits (6,7), early intervention is ideal.

Although no interventional studies in Ecuador have specifically targeted preschool-aged children, 1 study in neighboring Colombia found that a behavioral intervention could successfully improve knowledge, attitudes, and habits relevant to healthy eating and physical activity, for both children and their parents or guardians in Bogotá (8). Globally, numerous studies have evaluated behavioral interventions to improve nutrition and physical activity habits among young children (9–13).

In response to the growing problem of childhood overweight and obesity in Ecuador, we developed a context-specific educational and behavioral intervention that aimed to improve nutrition and physical activity habits. The objective of this study was to implement and evaluate this intervention at municipal preschools in Cuenca, Ecuador.

## Methods

Our study consisted of a 3-month pilot intervention (PI) and a 7-month enhanced intervention (EI) in 2 sequential school years among 2 different groups of children at all 9 municipal preschools in the city of Cuenca (Figure). Cuenca is a city in Ecuador’s southern Andean highland region with about 500,000 people, mostly of *mestizo* race, in its greater metropolitan area (14). The inclusion criteria for children were to attend a municipal preschool in Cuenca and be 3–4 years of age. Before this study, no official policies or recommendations existed regarding the nutritional content of food, meal/snack frequency, and physical activity in municipal preschools. The only practice in place was to have a nutritionist from the municipal government provide weekly menus to the preschools. Signed informed consent was obtained from all parents or guardians. In the PI and EI groups, 8 and 7 children, respectively, had a parent or guardian refuse participation in the study. This study (ClinicalTrials.gov identifier: NCT02451410) was funded by the Dirección de Investigación, Universidad de Cuenca, and was approved by the institutional review board of Universidad San Francisco de Quito (#2015–052E).

**Figure:**
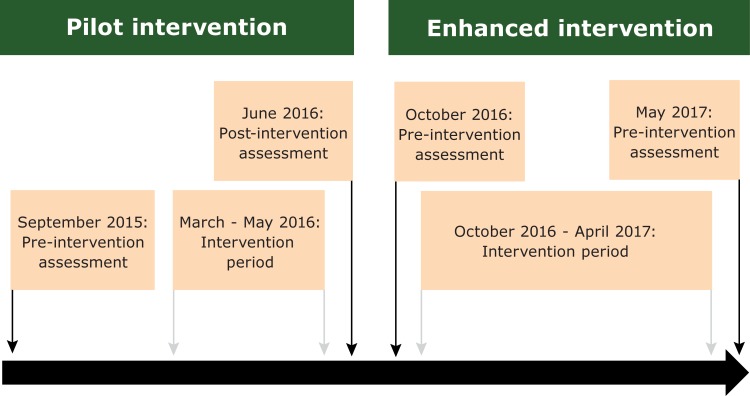
Study timeline of the pilot intervention and enhanced intervention, Preschool Nutrition and Activity Study, Cuenca, Ecuador, 2015 – 2017. The figure represents the study timeline of the pilot intervention (PI) and enhanced intervention (EI) which occurred sequentially in the 2015–2016 and 2016–2017 school years. For the PI, the lag time between the pre-intervention assessment and the start of the intervention resulted from personnel changes at the Ministry of Education, which necessitated their re-review of the proposed intervention.

The intervention was based in Social Cognitive Theory (15), and was developed after conducting a series of focus groups with parents, teachers, and school administrators, direct observation of environments in the preschools, and surveys. The activities of the intervention were focused on 3 principal goals: 1) drinking water instead of sugar-sweetened beverages; 2) eating fruits and vegetables at snack times; and 3) engaging in physical activity rather than screen time during free time. The activities centered around a story line of 4 fictional characters: Anita and Julián, both preschool-aged children, a turtle named George, and a hummingbird named Moti. Teachers underwent training before the intervention and received additional monthly training for 8 months for the PI and 6 months for the EI. Training not only consisted of how to deliver the intervention but also included training on nutrition and physical activity appropriate for preschoolers, such as age-appropriate serving sizes, making healthy choices, food hygiene, and identifying and preventing eating difficulties. Research staff visited preschools weekly to ensure that the intervention was being implemented appropriately.

The PI consisted of incorporating new activities into the existing school curriculum, such as “motor stories” where children heard about different activities in a story, such as growing and eating fruits and vegetables, and after the story the children participated in these activities. These daily activities focused on underscoring the 3 principal goals of the intervention in a fun way, using puppets, replica models of foods, pre-recorded songs, and pop-up books. One hour per day was dedicated to specific motor activities that were part of the intervention, which included games and activities where children had some kind of structured group physical activity. Classrooms were also provided with personal water bottles for the children and an organizer for them. To encourage behavior change, teachers were also given a board displaying the children’s names and traffic light stickers to indicate the adequacy of children’s observed drinking, eating, and physical activity habits throughout the day. This board was displayed in the classroom and was used as a means of encouraging children to strive to develop good habits, with care not to single out any particular student.

In addition to the content of the PI, the EI included activities for children to do with their parents; these were a continuation of school-based activities. For example, in school, children learned how to play certain games that included physical activity, and then parents were shown how to continue these activities at home. Parents were trained by teachers in workshops, and information was also provided in a workbook. In the workbook, parents provided evidence of completion of a given activity, and for each activity completed, children and their parents were given a different magnet for their refrigerator with images of the theme characters engaging in healthy activities.

The pre- and post-intervention assessments for the PI and EI groups consisted of questionnaires to the parent or guardian about demographic information and children’s at-home nutrition habits, sedentary time, and anthropometry. Trained nutritionists administered the surveys and conducted the anthropometry. The main outcomes of interest were change in BMI-for-age *z *score and proportion of children who were categorized as at risk for overweight or had overweight or obesity, and at-home changes in consumption of water and sugar-sweetened beverages, consumption of fruits and vegetables for snacks, and screen time.

To determine nutritional status, body weight was measured by using a digital scale (Seca) calibrated to the nearest 0.1 kg, with the children wearing light clothing. Height was measured without shoes by using a portable stadiometer (Seca) with a precision of 1 mm. Waist circumference was measured by using a nonelastic flexible measuring tape (Seca) midway between the lowest rib margin and the iliac crest with the child in a standing position and was recorded to the nearest 1 mm. Body weight, height, and waist circumference were measured twice and the average of the 2 values was determined. Nutritional status was defined by using the 2006 World Health Organization growth reference curves for children aged 0 to 60 months (16): underweight (weight-for-age *z *score below −2 standard deviations [SDs] of the median), overweight (weight-for-age or BMI-for-age *z *score between +2 and +3 SDs), obese (weight-for-age or BMI-for-age *z *score above +3 SDs), and stunted (height-for-age *z *score below −2 SDs). We also used the classification of “risk of overweight” (3), defined as weight-for-age or BMI-for-age *z *score between +1 and +2 SDs, and thus defined normal weight as having a weight-for-age or BMI-for-age *z *score between −2 and +1 SDs.

For at-home habits, parents were asked about their child’s typical weekly consumption frequency in the past 30 days (ie, never, less than once a week, 1–3 days per week, 4–6 days per week, and every day) of water, sugar-sweetened beverages, fruit for snacks, and vegetables for snacks, providing examples for the last 3 groups. For water and sugar-sweetened beverage consumption, parents were asked about their child’s habits separately for snacks (between meals) and during meals, which were combined to determine total consumption. The sedentary time component of the questionnaire asked about typical daily hours of screen time in the past 30 days, asking separately for weekday and weekend habits, with separate questions for television, movies, computer or cellular phone or tablet, and videogames. We recategorized water and sugar-sweetened beverage consumption as every day (vs all other categories); we combined fruit and vegetable consumption for snack, and recategorized the variable as every day (vs all other categories); and we recategorized weekday and weekend screen time as 2 hours or more (vs 1 or <1 hour) daily (17). In an effort to better characterize the demographics of our population, we also measured socioeconomic status (SES) (18) and household food insecurity (19) at baseline for the EI group. Because this decision was made after the PI ended, no such data were available for that group.

For the statistical analysis, we first computed descriptive statistics (frequencies, or means with SDs) for the PI and EI groups, and bivariate statistical tests (*t* tests for continuous variables; χ^2 ^tests or Fisher exact tests for categorical variables) to compare all variables by group. Next, we used generalized estimating equations (GEEs) to compute within-group pre–post differences for the outcomes of interest for both the PI and EI groups, and to compare the PI and EI groups, all unadjusted for any covariates. GEEs were chosen to account for a nested effect of repeated measures among children clustered in 1 of 9 preschools. As a sensitivity analysis, we re-ran GEE models for outcomes that were significantly different between the PI and EI group, adjusting for any baseline variables that were different between groups (at a *P* < .10). All analyses were conducted in SAS 9.4 (SAS Institute Inc., Cary, NC) and the significance level was set at a two-sided α of 0.05.

## Results

Overall 155 children were enrolled in the 3-month PI with 132 children completing the follow-up assessment, and in the next school year 152 children were enrolled in the 7-month EI with 144 completing the follow-up assessment.

Mean age in the PI and EI groups was 40.4 months and 42.8 months, respectively (*P* < .001 between groups). Slightly more than half of the children were boys in both the PI (54.2%) and EI (52.0%) groups. For both groups, more than 80% of baseline interviews were conducted with the child’s mother. In the EI, nearly all children had middle SES households (94.8%), but more than half of households (58.5%) had some degree of household food insecurity (Table 1).

**Table 1:** Baseline Characteristics of the Pilot and Enhanced Intervention Groups, Preschool Nutrition and Activity Study, Cuenca, Ecuador, 2015–2017

Characteristic^a^	Pilot Intervention (PI) Group N=155	Enhanced Intervention (EI) Group N=152	*P* value
**Age in months, mean (SD)**	40.4 (3.1)	42.8 (3.8)	<.001^b^
**Sex**			
Male	84 (54.2)	79 (52.0)	.70^c^
**Household socioeconomic status**			
Low	— d	5 (3.3)	—
Middle-low	— d	60 (39.5)	—
Middle	— d	48 (31.6)	—
Middle-high	— d	36 (23.7)	—
High	— d	3 (2.0)	—
**Household food insecurity**			
None	— d	64 (41.5)	—
Mild	— d	71 (46.7)	—
Moderate	— d	16 (10.5)	—
Severe	— d	2 (1.3)	—
**Person answering baseline questionnaire**			
Mother	128 (82.6)	125 (82.2)	.76^e^
Father	22 (14.2)	24 (15.8)	–
Other	5 (3.2)	3 (2.0)	–
**Anthropometry**			
Height in cm, mean (SD)	94.2 (4.3)	95.2 (4.4)	.78^b^
Weight in kg, mean (SD)	14.5 (1.7)	14.6 (1.8)	.31^b^
Waist circumference in cm, mean (SD)	50.5 (3.0)	50.1 (2.9)	.31^b^
BMI, mean (SD)	16.3 (1.2)	16.1 (1.1)	.03^b^
BMI-for-age (*z *score)			.05^b^
Mean (SD)	0.65 (0.82)	0.47 (0.79)	–
Median (range)	0.63 (−1.62 to 2.87)	0.41 (−1.39 to 2.78)	–
**Stunting**	24 (15.6)	22 (14.6)	.80^c^
**Child nutritional status**			
Underweight	3 (2.0)	3 (2.0)	.88^e^
Normal	138 (89.6)	139 (92.1)	–
At risk for overweight	12 (7.8)	8 (5.3)	–
Overweight^f^	1 (0.7)	1 (0.7)	–
**Daily water consumption**	116 (75.8)	112 (73.7)	.67^c^
**Daily sugar-sweetened beverage consumption**	50 (32.3)	34 (22.4)	.05^c^
**Daily fruit and vegetable consumption for snacks**	25 (16.1)	26 (17.1)	.82^c^
**Screen time (≥2 h)**			
Weekday	140 (90.3)	132 (87.4)	.42^c^
Weekend	132 (85.2)	135 (88.8)	.34^c^

Abbreviations: BMI, body mass index; SD, standard deviation.

a Data are reported as n (%) unless otherwise noted.

b *t* test.

c χ^2^ test.

d Not measured for the PI group.

e Fisher exact test.

f No children had obesity at baseline.

BMI-for-age *z *score was borderline significantly higher in the PI group compared with the EI group (mean 0.64 vs 0.47; *P* = .05), but there was no significant difference between groups for nutritional status, with roughly 90% of children at normal weight, 6% categorized as at risk for overweight, and <1% overweight. At-home habits were overall similar in the PI and EI groups. Roughly three-quarters of parents reported that their child drank water daily (75.8% PI, 73.7% EI). About one-sixth reported that their child had fruits and vegetables daily for snacks (16.1% PI, 17.1% EI). Most children had at least 2 hours of daily screen time during the week (90.3% PI, 87.4% EI) and during the weekend (85.2% PI, 88.8% EI). There was, however, a borderline significant difference (*P* = .05) for higher daily sugar-sweetened beverage consumption for the PI group (32.3%) compared with the EI group (22.4%) (Table 1).

For the PI group, significant pre- and post-intervention differences were found for mean BMI-for-age *z *score (+0.15; *P* = .002) and sugar-sweetened beverage consumption (−23.2%; *P* < .001). There was a borderline significant increase in the proportion of children categorized as at risk of overweight, or who had overweight or obesity (+3.9%; *P* = .08). For the EI group, significant pre- and post-intervention differences existed for mean BMI-for-age *z *score (−0.11; *P* = .003), water consumption (+8.3%; *P* = .04), sugar-sweetened beverage consumption (−16.8%; *P* < .001), fruit and vegetable consumption (+21.8%; *P* < .001), and weekend screen time of at least 2 hours daily (−7.6%; *P* = .03). At baseline, 6.1% of children were categorized as at risk for overweight, or had overweight or obesity, but at follow-up no children were in this category, representing a 6.1% reduction. No children in this category were lost to follow-up.

When comparing the changes in the PI versus EI groups, there were significant differences in mean BMI-for-age *z *score (−0.25; *P* < .001) and fruit and vegetable consumption (+15.9%; *P* = .01) (Table 2). We conducted additional analyses to determine if the association favoring the EI group (vs the PI group) for BMI-for-age *z *score and daily fruit and vegetable consumption remained significant after adjusting for baseline differences between groups (data not shown). In the first model, with BMI-for-age *z *score as the dependent variable and adjusting for age, and baseline sugar-sweetened beverage consumption, the difference in means was −0.28 (95% confidence interval [CI] −0.17, −0.40; *P* < .001). In the second model with fruit and vegetable consumption as the dependent variable and adjusting for age, BMI-for-age *z *score, and baseline sugar-sweetened beverage consumption, the difference was +16.4% (95% CI 3.5–29.4; *P* = .013).

**Table 2:** Changes from Baseline in Anthropometry and Habits at Home for Pilot and Enhanced Intervention Groups, and Comparisons Between Groups, Preschool Nutrition and Activity Study, Cuenca, Ecuador, 2015–2017

Measure^a^	Pilot Intervention (PI)			Enhanced Intervention (EI)			PI vs EI
	Pre-intervention Mean *z* Score (SD) n=155	Post-intervention Mean *z* Score (SD) n=132	Pre–post difference (95% CI) [*P* Value^b^]	Pre-intervention Mean *z* Score (SD) n=152	Post-intervention Mean *z* Score (SD) n=144	Pre–post difference (95% CI) [*P* value^b^]	Difference in pre–post differences (95% CI) [*P* value^b^]
**Anthropometry**							
**BMI-for-age** ***z* score**	0.65 (0.82)	0.79 (0.85)	0.15 (0.05 to 0.24) [.002]	0.47 (0.79)	0.36 (0.77)	−0.11 (−0.04, −0.17) [.003]	−0.25 (−0.37, −0.14) [<.001]
**At risk of overweight, or were overweight or obese**	13 (8.6)	16 (12.5)	+3.9 (−0.4 to 8.2) [.08]	9 (6.1)	0 (0.0)	−6.1 (NA^c^) [NA^c^]	−10.0 (NA^c^) [NA^c^]
**Habits at home**							
**Daily water consumption**	116 (75.8)	101 (76.5)	+0.7 (−8.0 to 9.4) [.88]	112 (73.7)	118 (81.9)	+8.3 (0.4, 16.1) [.04]	7.6 (−4.2, 19.3) [.21]
**Daily sugar- sweetened beverage consumption**	50 (32.3)	12 (9.1)	−23.2 (−14.8 to −31.5) [<.001]	34 (22.4)	8 (5.6)	−16.8 (−9.1, −24.6) [<.001]	6.4 (−5.1, 17.8) [.28]
**Daily fruit and vegetable consumption for snacks**	25 (16.1)	29 (22.0)	+5.8 (−2.7 to 14.4) [.18]	26 (17.1)	56 (38.9)	+21.8 (12.9, 30.7) [<.001]	15.9 (3.7, 28.2) [.01]
**Daily screen time ≥2 h**							
Weekday	140 (90.3)	118 (89.4)	−0.9 (−7.1 to 5.3) [.77]	132 (87.4)	126 (87.5)	+0.1 (−6.8, 7.0) [.98]	1.0 (−8.3, 10.3) [.83]
Weekend	132 (85.2)	112 (84.9)	−0.3 (−7.7 to 7.1) [.93]	135 (88.8)	117 (81.3)	−7.6 (−14.5, −0.6) [.03]	−7.3 (−17.4, 2.9) [.16]

Abbreviations: BMI, body mass index; CI, confidence interval; SD, standard deviation.

a Data are reported as n (%) unless otherwise noted. Not all percentages are based on the denominator indicated in the column head, because of listwise deletion of missing data. Differences may not sum due to rounding.

b Unadjusted generalized estimating equation with a nested effect (repeated measures among children who were within 1 of 9 preschools).

c Not computed because of 0 cell.

## Discussion

We observed a beneficial effect of our intervention on reducing parent-reported daily sugar-sweetened beverage consumption in both the PI and EI, but with the EI we also observed beneficial effects on daily water consumption, daily fruit and vegetable consumption for snacks, and weekend screen time, as well as BMI-for-age *z *score. When comparing the PI and EI, there were significant differences between interventions for their effect on BMI-for-age *z *score and fruit and vegetable consumption. Longer-term follow-up is required to determine if the EI had a lasting effect that translated to reduced prevalence of overweight and obesity in school-age years.

Before conducting this study, nutrition and physical activity were not addressed in the preschool curriculum, nor were there any policies in place regarding the provision of water, healthy foods, and physical activity in municipal preschools. In Latin America, undernutrition has been a historic focus of public health interventions; however, as the region changes, especially in terms of SES, urbanization, and food and drink consumption, interventions to prevent and reduce childhood obesity need to be made a public health priority (20). The Ecuadorian Ministry of Health has published guides to promote nutrition and physical activity among children, including preschool-aged children (21), yet despite the availability of these materials, no specific programs have been implemented, and the prevalence of childhood obesity continues to rise (3). Although this intervention provides municipal preschools with an evidence-based tool to integrate into their curriculum, education policy still needs to be changed. For example, preschools should adhere to uniform policies with specific standards for nutrition, including having healthy meals and snacks with age-appropriate portions and ensuring access to drinking water, and physical activity, including increasing outdoor time and physical activity and limiting sedentary and entertainment screen time (22–24).

Globally, trials of school-based interventions aiming to improve nutrition and physical activity habits have included a wide range of methodologic approaches (9–13). Ideally, to determine the efficacy of an intervention, there would be a control group for comparison that received some type of “usual care.” We did not include a control group with any intervention because similar types of interventions have been beneficial in other settings with minimal risk (9–13) and there was no usual established nutrition or physical activity curriculum in schools. It is very difficult, if not impossible, to tease out the effect of an intervention from a single group using pre- and post-testing, and for this reason, we compared the EI with the PI. This helped provide additional evidence supporting a positive effect of the intervention. However, despite comparing these 2 groups, we still could not ensure complete temporal control. Both the PI and EI were done at the same preschools and it is possible that the teachers did a better job of implementing the intervention in the second year, when the EI was done. Although the greater effect of the EI on parent-reported measures might have been related to the direct participation of parents in the study versus the PI, which was only school-based, it is reassuring that there was also a positive effect on objectively measured BMI-for-age *z *score. Another benefit of the EI was that we were able to identify challenges with the PI and resolve these for the EI. For example, to improve acceptability, we invited the teachers to provide feedback on activities from the PI and help in planning and modifying activities for the EI.

Some additional limitations should be considered with this study: First, we were not able to objectively measure at-home nutrition habits and sedentary time. All data were self-reported, mostly by mothers, so it is not clear to what extent these self-reports coincided with actual behavior, as social desirability bias may have affected estimates. With the exception of some studies that have objectively measured physical activity, most nutrition and sedentary time habits have been self-reported by parents (9–13). Additionally, lack of sensitivity of the self-reported measures is another concern; for example, the response “daily” likely encompassed a wide range of habits. However, this was a trade-off with simplicity. Second, although teachers were trained in the curriculum, we did not train teachers to role model healthy eating and physical activity, which, based on prior research (25–27), is an important consideration for further improving the intervention. Third, the intervention had limited effects on screen time. Additional evaluation is needed to better ascertain if the intervention should be modified to include more emphasis on physical activity, or if alternative methods need to be employed for measuring these variables, which remain very difficult to measure among preschoolers, even objectively (28). Finally, there was differential loss to follow-up between groups (14.8% in the PI group and 5.3% in the EI group). As this was likely caused by the later June assessment of the PI group coinciding with a school holiday, it seems unlikely to have strongly biased our results.

The results of our study support the idea that preschool-based nutrition and physical activity intervention in Cuenca is indeed feasible and beneficial to preschoolers, and should be incorporated into the curriculum of municipal preschools. Additional follow-up is needed to determine if the intervention had long-term effects and future evaluation efforts should test implementation of the intervention outside of the municipal preschool system in Cuenca, and possibly elsewhere in Ecuador. Additional research is needed to further enhance the intervention, and determine how to modify it to be appropriate for other settings, such as outside of the municipal preschool system and in the coastal and Amazon regions of Ecuador, given differences in culture, built environment, and types of available food and drink.
